# Case Report of Traumatic Uterine Rupture in a Multigravida Woman with Emergency Department Cesarean Section

**DOI:** 10.5811/cpcem.2020.8.48388

**Published:** 2020-10-09

**Authors:** Krista Shaw Wiese, Stacey Ernest, W. Seth Dukes

**Affiliations:** Loma Linda University, Department of Emergency Medicine, Loma Linda, California

**Keywords:** traumatic uterine rupture, emergency cesarean section, trauma in pregnancy

## Abstract

**Introduction:**

Uterine ruptures in blunt trauma are an extremely rare complication. Furthermore, while perimortem cesarean sections in cardiac arrest patients are a well-established practice in emergency medicine, cesarean sections in the emergency department are rarely performed on non-arresting patients.

**Case Report:**

A multigravida woman at approximately 24 weeks gestation presented as a transfer from an outside hospital after a motor vehicle collision. Upon arriving to our facility, she underwent an emergency cesarean section in the trauma bay and was found to have a uterine rupture with the fetus free floating in the right upper quadrant of the abdomen.

**Conclusion:**

Uterine rupture is a rare but important complication of blunt abdominal trauma in pregnant patients. Resuscitative cesarean sections may be necessary for favorable outcomes. A well prepared and diversified team was essential to maternal survival.

## INTRODUCTION

There are guidelines in emergency medicine regarding timelines for perimortem cesarean delivery (PMCD) in the case of maternal cardiac arrest. The American Heart Association recommends that PMCD should be initiated after four minutes of failure of resuscitative efforts with a goal of delivery within five minutes (the four- to five-minute rule) of initiation of resuscitative efforts in the case of a pregnant patient with estimated fetal gestation greater then 24 weeks.^1^ Current case reports describe PMCD in the setting of a patient who has already undergone cardiac arrest. Less commonly described is a patient who has not undergone cardiac arrest but receives a cesarean section in the emergency department (ED) as a resuscitative measure. A well prepared and diversified team is essential to make this decision upon patient presentation. Herein, we present a multiparous woman who was involved in a motor vehicle collision, initially presented to an outside hospital, and was ultimately transferred to our facility. She underwent an emergent cesarean section in our trauma bay and was found to have a uterine rupture with the fetus free floating in the right upper quadrant of the abdomen.

## CASE REPORT

A 30-year-old female gravida 3, para 2 with an intrauterine pregnancy at approximately 24 weeks was transferred from an outside hospital and was activated as a trauma prior to arrival to our facility. The patient was the restrained driver of her vehicle, which had been traveling approximately 70 miles per hour. She was involved in a multivehicle accident, which occurred two hours prior to her presentation to our facility. The patient was not initially transported by emergency medical services but instead went by private auto to the outside facility. On presentation to that hospital the patient was noted to have abrasions with underlying ecchymoses across the gravid lower abdomen and the left side of the chest and neck consistent in patterning with where her seatbelt would have been positioned. She also complained of abdominal pain.

Vitals were taken and she was hypotensive with maternal blood pressures 90s/40s millimeters mercury (mm Hg), and tachycardic with heart rate in the 120+ beats per minute (bpm) range. Fetal heart tones were in the 80s bpm. Point-of-care ultrasound was positive for free fluid in the abdomen. Resuscitative efforts were started. As a Level I trauma center, our institution has an agreement with the non-trauma designated hospitals in our area. If they receive a trauma patient, they inform our trauma center that they will be transferring the patient and we automatically accept the patient to expedite the transfer process for what we call a continuation of trauma. For this particular patient a continuation of trauma was initiated. Prior to presentation to our facility, the ED, obstetrics, acute care surgery (ACS), and neonatal intensive care unit (NICU) teams gathered in the trauma bay as one cohesive unit to formulate several contingency plans based on the patient’s presentation (see table).

Upon initial presentation her vitals were blood pressure 127/97 mm Hg, heart rate 145 bpm, respiratory rate 28 breaths per minute, and she was saturating 100% on room air. Her Glasgow Coma Scale was 14 (eye = 3; verbal = 5; motor = six). She was noted to have abrasions and ecchymoses across her gravid lower abdomen, left chest and neck, which were consistent in patterning with an overlying seatbelt. Vaginal bleeding was also noted. The obstetrics team performed a point-of-care ultrasound with fetal heart rates in the 50s bpm, and the decision was made to proceed with emergent cesarean delivery. The ED senior resident intubated the patient and managed medications. Emergent cesarean section was performed via a low transverse incision by the obstetrics team.

Manual evaluation of the lower uterine segment revealed a large defect, and no fetus was palpable in the uterus. Further evaluation of the abdomen revealed the fetus was free floating in the right upper quadrant. The fetus was delivered and handed off to waiting NICU staff. A large, lower uterine segment defect was noted extending in the midline to the cervix. The hysterotomy was closed with adequate hemostasis, and the decision was made to proceed to the operating room for further evaluation. Upon initial assessment of the fetus, there was no pulse. The fetus was intubated and chest compressions were begun. Standard Neonatal Advanced Life Support protocol was initiated. Patient remained in asystole and time of death was called after 22 minutes of resuscitation.

The mother was transferred to the operating room, and the ACS team performed an exploratory laparotomy through the Pfannenstiel incision made in the trauma bay. Minor bleeding near the right uterine artery was found and controlled. No liver, spleen, stomach, small or large bowel injuries were appreciated. Patient received four units of packed red blood cells, three units of fresh frozen plasma, and one unit of platelets between the ED and operating room. Postoperatively, the patient underwent computed tomography (CT) of the head, cervical/lumbar/thoracic spine, chest, and abdomen, and CT angiography of the neck. No further injuries were discovered. The patient was extubated on postoperative day (POD) zero and was downgraded from the surgical intensive care unit on POD one.

CPC-EM Capsule

What do we already know about this clinical entity?

*There are clear guidelines in emergency medicine regarding timelines for perimortem cesarean delivery in the case of maternal cardiac arrest*.

What makes this presentation of disease reportable?

*Our case report highlights the usefulness of performing a cesarean section as a resuscitative effort on a non-arresting, pregnant trauma patient*.

What is the major learning point?

*A pregnant trauma patient with a uterine rupture may require a cesarean section in the emergency department before she goes into cardiac arrest*.

How might this improve emergency medicine practice?

*Awareness of a rare complication of uterine rupture will alert providers to consider cesarean section as a resuscitative measure in the trauma bay*.

On POD two she had persistent unexplained tachycardia. CT pulmonary angiography was obtained, which showed subsegmental pulmonary embolism. She was treated with a heparin drip and transitioned to enoxaparin and then rivaroxaban. She was discharged on POD six. She was readmitted three weeks after discharge for acute anemia in the setting of anticoagulation. The patient was transfused three units of red blood cells and discharged again two days later. At her eight-week postpartum visit she was doing well with no major issues.

## DISCUSSION

This case presented two unique features in the traumatic resuscitation of a pregnant patient, including blunt trauma causing uterine rupture, and an ED cesarean section in a non-arresting patient as a resuscit ation effort. Post-trauma uterine rupture is an extremely rare complication (0.6% of all trauma-related maternal injuries). They are seen more frequently in women who have a previously scarred uterus or with direct abdominal impact in the latter half of the pregnancy.^2^ Maternal mortality does occur, but fetal mortality is almost universal.^3^ Suspected uterine rupture with maternal and/or fetal compromise should prompt urgent resuscitative laparotomy.^4^ Our patient followed a similar pattern with a gestational age of approximately 24 weeks, history of previous uterine scarring, seatbelt sign overlying the lower uterine segment causing direct abdominal impact and ultimately fetal death. Surgical intervention consisting of exploratory laparotomy is often required.

Our patient represents a unique case in which surgical intervention was initiated within the ED. The case further demonstrates surgical intervention initiated on a non-arresting, pregnant patient. In cases of maternal cardiac arrest, a well defined practice pattern of performing a perimortem cesarean section has been in place since 1986. This protocol includes cesarean section four minutes after maternal pulse ceases in women with an estimated fetal age greater then 24 weeks or fundal height two or more fingerbreadths above the umbilicus.^5^ Cesarean sections in the ED are much less frequently performed in non-arresting patients. Per our literature review, we could not find any other documented cases of patients undergoing a cesarean section in the ED in a mother who had not already gone into cardiac arrest.

## CONCLUSION

Uterine rupture is an extremely rare complication of maternal trauma but should be a consideration when approaching a pregnant trauma patient, especially if there is evidence of direct blunt trauma and concerning vital signs. Even if these patients are not in cardiac arrest, they may require an emergent cesarean section in the ED. Furthermore, teamwork between a multidisciplinary trauma team can be essential in achieving good outcomes. We firmly believe that in this case, the maternal outcome was favorable because of the rapid decision-making and team effort of four distinct specialties prior to patient arrival and during the resuscitation. Although advanced planning is not always possible in the ED, whenever feasible it is essential to agree upon a number of contingency plans especially when multiple teams are involved in a complex trauma patient such as this one.

## Figures and Tables

**Table:** Varying plans for differing patient presentations agreed upon by team leaders prior to patient presentation.

	Situation	Plan
1	Mother and fetus are stable.	Acute care surgery will proceed with standard primary trauma survey.
2	Mother unstable but not in cardiac arrest and fetus unstable.	Obstetrics will perform cesarean section in the emergency department.
3	Mother in cardiac arrest and fetus unstable.	Emergency department senior resident will perform perimortem cesarean section.
